# Cognitive Improvement and Chronic Pain Modulation with Vortioxetine in a Case of Cranial Anesthetic Migration: A Clinical Case

**DOI:** 10.1192/j.eurpsy.2026.11410

**Published:** 2026-07-31

**Authors:** M. S. Molina, E. A. Sánchez, C. D. Mayoral, J. G. Bonaque

**Affiliations:** 1 Psychiatry, Hospital Universitario Príncipe de Asturias; 2 Psychiatry, Hospital Blua Sanitas Valdebebas, Madrid, Spain

## Abstract

**Introduction:**

The case of a 50-year-old woman with depressive, anxious, and cognitive symptoms, along with chronic pain, following a singular neurological event: the cranial migration of an anesthetic. This comorbidity, characterized by the complex interrelation between affective symptoms, potential neurological damage, and pain symptomatology, poses a significant diagnostic and therapeutic challenge.

**Objectives:**

To describe the clinical evolution in a case of neuropsychiatric comorbidity and chronic pain treated with vortioxetine, based on its multimodal pharmacological profile, to address mood symptoms, cognitive alterations, and pain modulation.

**Methods:**

The patient started treatment with vortioxetine and was followed up at a mental health center. The therapeutic response was evaluated through the clinical evolution of depressive and anxious symptoms, cognitive complaints, and the pain symptomatology reported by the patient during follow-up consultations.

**Results:**

After starting treatment with vortioxetine, the patient experienced a progressive and significant improvement in her mood and anxiety complaints. Notably, her cognitive difficulties, including memory, processing speed, and concentration, improved markedly, allowing for greater functionality in her daily life. The patient also reported a significant reduction in her chronic pain symptoms, suggesting a potential analgesic effect of vortioxetine in this clinical context, as described in recent literature.

**Conclusions:**

This clinical case demonstrates the effectiveness of vortioxetine in a complex scenario of neuropsychiatric comorbidity. The improvement observed in the patient directly aligns with the scientific evidence supporting vortioxetine’s dual action on affective symptoms, cognitive functions, and pain modulation, a key therapeutic benefit in patients with depression and comorbidities. The results suggest that vortioxetine is a valuable treatment option for addressing the complex interrelation of these symptoms.

**Disclosure of Interest:**

None Declared

